# Supplementary figures and images for: Molecular detection of avian hepatitis E virus (Orthohepevirus B) in chickens, ducks, geese, and western capercaillies in Poland
Source: PLoS One. 2022 Jun 23;17(6):e0269854. doi: 10.1371/journal.pone.0269854 (PMC9223332; doi:10.1371/journal.pone.0269854)

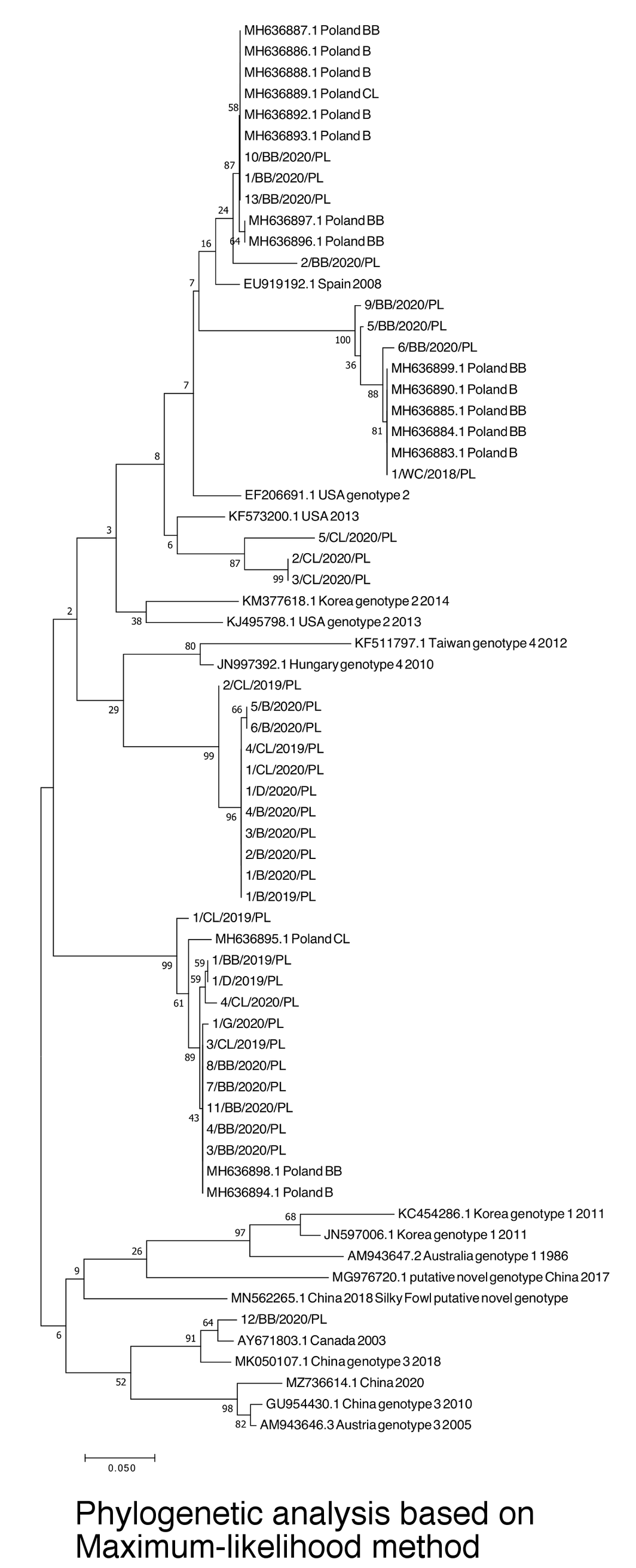

Supplement: S1 Fig — (TIF) [file pone.0269854.s001.tif]

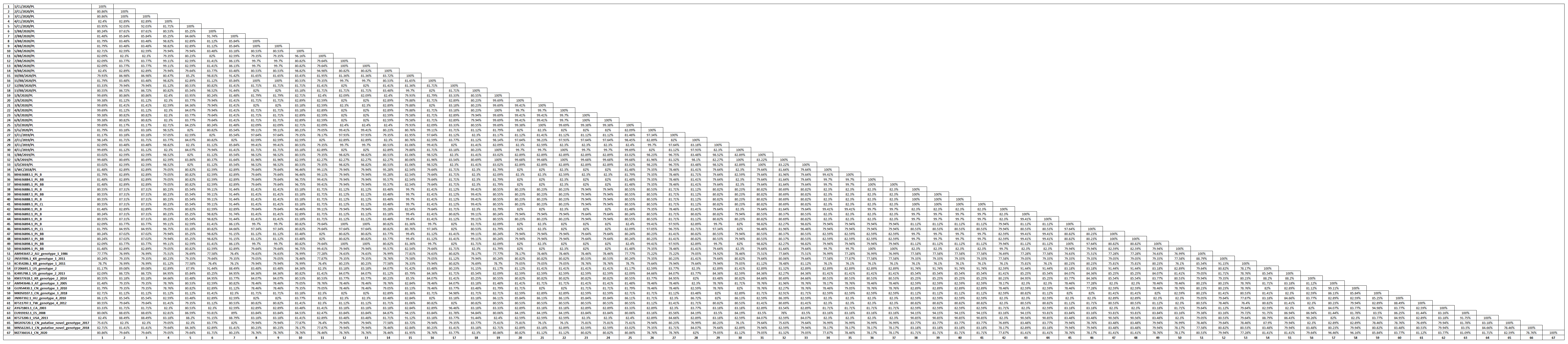

Supplement: S1 Table — (TIF) [file pone.0269854.s002.tif]

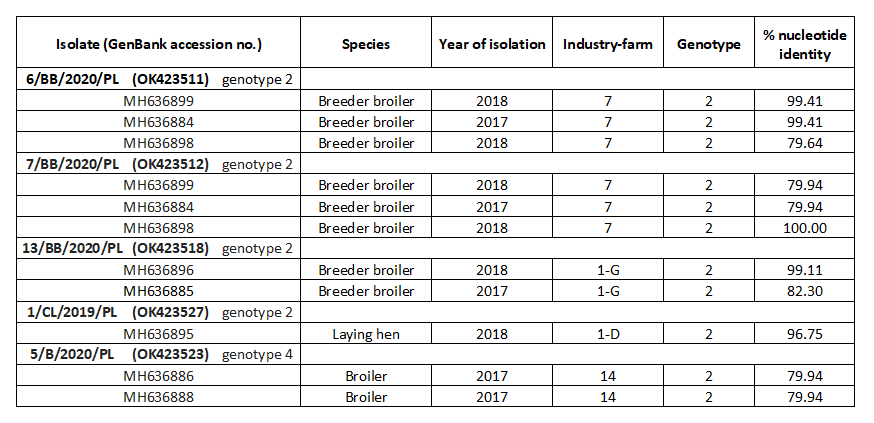

Supplement: S2 Table — (TIF) [file pone.0269854.s003.tif]
