# Supplementary material for: Molecular detection of avian hepatitis E virus (Orthohepevirus B) in chickens, ducks, geese, and western capercaillies in Poland
Source: PLoS One. 2022 Jun 23;17(6):e0269854. doi: 10.1371/journal.pone.0269854 (PMC9223332; doi:10.1371/journal.pone.0269854)
Supplement: S1 Data — (PDF) [file pone.0269854.s004.pdf]

Table Analyzed	Data 1
Column B	tested aHEV CL
vs.	vs,
Column A	infected aHEV CL
Unpaired t test with Welch's correction	
P value	0,1236
P value summary	ns
Significantly different (P < 0.05)?	No
One- or two-tailed P value?	Two-tailed
Welch-corrected t, df	t=1,625, df=16,19
How big is the difference?	
Mean of column A	37,67
Mean of column B	31,1
Difference between means (B - A) ± SEM	-6,567 ± 4,042
95% confidence interval	-15,13 to 1,994
R squared (eta squared)	0,1401
F test to compare variances	
F, DFn, Dfd	2,010, 39, 8
P value	0,2991
P value summary	ns
Significantly different (P < 0.05)?	No
Data analyzed	
Sample size, column A	9
Sample size, column B	40

Table Analyzed	Data 1	Table Analyzed	
Column D	tested aHEV BB	Column F	
vs.	vs,	vs.	
Column C	infected aHEV BB	Column E	
Unpaired t test with Welch's correction		Unpaired t test with Welch's corr	
P value	0,232	P value	
P value summary	ns	P value summary	
Significantly different (P <	No	Significantly different (P < 0.05)'	
One- or two-tailed P valu	Two-tailed	One- or two-tailed P value?	
Welch-corrected t, df	t=1,227, df=23,82	Welch-corrected t, df	
How big is the difference?		How big is the difference?	
Mean of column C	41,71	Mean of column E	
Mean of column D	35,8	Mean of column F	
Difference between mear	-5,914 ± 4,822	Difference between means (F -	
95% confidence interval	-15,87 to 4,041	95% confidence interval	
R squared (eta squared)	0,05941	R squared (eta squared)	
F test to compare variances		F test to compare variances	
F, DFn, Dfd	1,106, 39, 13	F, DFn, Dfd	
P value	0,8877	P value	
P value summary	ns	P value summary	
Significantly different (P <	No	Significantly different (P < 0.05)'	
Data analyzed		Data analyzed	
Sample size, column C	14	Sample size, column E	
Sample size, column D	40	Sample size, column F	

Data 1	Table Analyzed
tested Broiler	Column H
vs,	vs.
infected Broiler	Column G
rection	Unpaired t test with Welch's correction
0,4961	P value
ns	P value summary
No	Significantly different (P < 0.05)?
Two-tailed	One- or two-tailed P value?
t=0,7100, df=8,799	Welch-corrected t, df
	How big is the difference?
3,714	Mean of column G
4,122	Mean of column H
0,4082 ± 0,5749	Difference between means (H - G) ± SE
-0,8968 to 1,713	95% confidence interval
0,05419	R squared (eta squared)
	F test to compare variances
1,501, 48, 6	F, DFn, Dfd
0,6454	P value
ns	P value summary
No	Significantly different (P < 0.05)?
	Data analyzed
7	Sample size, column G
49	Sample size, column H

Data 1	Table Analyzed	Data 1
tested Duck	Column F	tested Broiler
vs,	vs.	vs,
infected Duck	Column E	infected Broiler
	Unpaired t test with Welch's correction	
0,7067	P value	0,4961
ns	P value summary	ns
No	Significantly different (P < 0.05)	No
Two-tailed	One- or two-tailed P value?	Two-tailed
t=0,4626, df=1,364	Welch-corrected t, df	t=0,7100, df=8,799
	How big is the difference?	
4	Mean of column E	3,714
5	Mean of column F	4,122
1,000 ± 2,162	Difference between means (F -	0,4082 ± 0,5749
-14,00 to 16,00	95% confidence interval	-0,8968 to 1,713
0,1356	R squared (eta squared)	0,05419
	F test to compare variances	
	F, DFn, Dfd	1,501, 48, 6
	P value	0,6454
	P value summary	ns
	Significantly different (P < 0.05)	No
	Data analyzed	
2	Sample size, column E	7
23	Sample size, column F	49

Table Analyzed	Data 1
Column J	tested Geese
vs.	vs,
Column I	infected Geese
Unpaired t test	
P value	0,3883
P value summary	ns
Significantly different (P < 0.05)	No
One- or two-tailed P value?	Two-tailed
t, df	t=0,8679, df=72
How big is the difference?	
Mean of column I	2
Mean of column J	5,699
Difference between means (J -	3,699 ± 4,261
95% confidence interval	-4,796 to 12,19
R squared (eta squared)	0,01035
F test to compare variances	
F, DFn, Dfd	
P value	
P value summary	
Significantly different (P < 0.05)?	
Data analyzed	
Sample size, column I	1
Sample size, column J	73
